# Racial Inequities and Access to COVID-19 Treatment

**DOI:** 10.1001/jamanetworkopen.2025.18459

**Published:** 2025-07-01

**Authors:** Rebecca Bromley-Dulfano, Michael L. Barnett

**Affiliations:** 1 Health Policy and Management, Harvard T. H. Chan School of Public Health, Boston, Massachusetts; 2 Stanford University School of Medicine, Stanford, California; 3 Division of General Internal Medicine and Primary Care, Department of Medicine, Brigham and Women’s Hospital, Boston, Massachusetts

## Abstract

**Importance:**

Timely outpatient treatment for COVID-19 reduces the risk of hospitalization and death in high-risk patients. However, large racial and ethnic inequities in treatment have been observed across the US. The relative contributions of care mechanisms that create barriers (eg, access to home tests or telemedicine) are unknown.

**Objective:**

To examine the relative contribution of structural and encounter-level factors associated with racial and ethnic disparities in oral antiviral prescribing for COVID-19.

**Design, Setting, and Participants:**

In this cross-sectional study using electronic health record data from a large northeastern health care system, medication order receipt was estimated across racial and ethnic minority groups. The sample comprised patients aged 18 years or older with documented positive COVID-19 test results from January 1, 2022, through January 31, 2024.

**Exposures:**

Race and ethnicity as recorded in patient electronic health records.

**Main Outcomes and Measures:**

The primary outcome was prescription orders of nirmatrelvir/ritonavir or molnupiravir within 7 days of a positive COVID-19 test result. Nested linear probability models were used, adjusting for clinical characteristics (age, sex, comorbidities, and contraindications), public health determinants (vaccination, insurance, and language), time fixed effects, and encounter-level characteristics (virtual care, diagnostic test type, and clinic site). The relative contribution of these factors to racial and ethnic prescription disparity was then partitioned.

**Results:**

The sample consisted of 201 964 patients with positive COVID-19 test results (mean [SD] age, 54.0 [18.2] years; 64.6% female; 0.1% identified as American Indian or Alaska Native, 3.7% as Black, 6.5% as Hispanic or Latino, 79.0% as White, 1.1% as other, and 4.7% as unknown race and ethnicity). Most were commercially insured (66.4%). Unadjusted prescription rates were 10.8 percentage points (95% CI, 9.7-11.8 percentage points) lower for Black compared with White patients and 9.8 percentage points (95% CI, 9.0-10.6 percentage points) lower for Latino patients. Black and Latino patients were less likely than White patients to be diagnosed with in-clinic antigen tests (−8.4 percentage points [95% CI, −9.2 to −7.5 percentage points] and −7.7 percentage points [95% CI, −8.3 to −7.0 percentage points], respectively), by home antigen tests (−10.3 percentage points [95% CI, −11.5 to −9.2 percentage points] and −14.1 percentage points [95% CI, −14.9 to −13.2 percentage points], respectively), or to have received care virtually (−16.2 percentage points [95% CI, −17.3 to −15.0 percentage points] and −18.4 percentage points [95% CI, −19.3 to −17.5 percentage points], respectively). Observable covariates fully accounted for the unadjusted prescription differences, with 53% of the racial and ethnic prescription gap for Black patients and 39% for Latino patients explained by encounter-level factors.

**Conclusions and Relevance:**

This cross-sectional study found that racial and ethnic disparities in COVID-19 treatment are substantial and partially explainable by encounter-level factors, particularly differences in diagnostic test type, virtual care access, and site of care. These findings suggest that targeted interventions, including expanding rapid test access and virtual care, may improve equity in outpatient COVID-19 treatment.

## Key Points

**Question:**

What is the association of upstream structural determinants vs encounter-level care delivery mechanisms with racial and ethnic disparities in outpatient COVID-19 antiviral prescriptions?

**Findings:**

In this cross-sectional study of 201 964 patients with a positive COVID-19 test result, approximately half of the disparity between Black and Latino patients and their White counterparts was explained by encounter-level factors, such as COVID-19 test type, virtual care use, and site of care.

**Meaning:**

These findings suggest that increasing access to rapid antigen and home tests, virtual care, and targeted interventions among clinics serving large percentages of Black and Latino patients may meaningfully improve equity in outpatient COVID-19 treatment.

## Teaser

This cross-sectional study examines whether structural and encounter-level factors are associated with racial and ethnic disparities in oral antiviral prescriptions for patients with a positive COVID-19 test result.

## Introduction

Timely outpatient treatment for COVID-19 infection is highly effective at reducing the risk of severe disease and hospitalization, particularly for patients at high risk for severe complications.^1,2,3^ In December 2021, under emergency use authorization, the oral therapies nirmatrelvir/ritonavir^4^ (hereafter, nirmatrelvir) and molnupiravir^5^ were approved for outpatient treatment in the US for individuals at high risk of progression to severe COVID-19. These therapies were subsidized by the federal government, at no cost to patients, with the hope of being relatively accessible compared with monoclonal SARS-COV-2 antibodies or intravenous remdesivir, which required access to an infusion center.^6,7,8^

Despite the availability and affordability of these medications, racial and ethnic and socioeconomic inequities in COVID-19 treatment access quickly emerged. Unfortunately, patients at the highest risk for severe COVID-19, particularly those with disproportionately lower incomes and from racial and ethnic minority groups, have been the least likely to receive treatment in the outpatient setting.^8,9,10,11,12,13^ This discrepancy represents a major public health challenge not only for COVID-19 treatment but also for disseminating future novel treatments in a pandemic setting.

The mechanisms behind these disparities may reflect different manifestations of structural racism, or the forms of racism created and upheld by institutions, policies, and entrenched societal norms that perpetuate the inequality of racial and ethnic minority people.^14^ These upstream structural determinants could act through various mechanisms. Differences in clinical risk or baseline health at presentation due to inequitable exposure to adverse social determinants of health, such as low access to food, housing, or economic security, might impact prescription likelihood through differential comorbidity prevalence or severity of COVID-19 disease. Through this framework, factors such as age at presentation, sex, comorbidities, and potential contraindications to treatment (hereafter collectively referred to as clinical characteristics) proxy longitudinal exposure to structural health determinants. These factors could influence the clinical encounter long before a patient presents for COVID-19 treatment. Similarly, inequities influenced by the local policy and public health environment, such as access to insurance coverage, vaccination patterns, and language barriers (hereafter collectively referred to as public health determinants), might also impact prescription trends through health care access, clinician bias, or health-seeking behavior. While all these sources of inequity crucially need to improve, they are not amenable to short-term intervention in a pandemic setting and unlikely to change without large-scale policy action over years.

On a more immediately actionable level, there are also clinical, encounter-level factors that could influence COVID-19 treatment, such as differential access to clinicians willing to prescribe antivirals or delays in presenting to care.^9,10,11,13,15^ Other health care delivery factors, such as inequitable access to virtual care or differential use of home tests, which can be costly but allow for point-of-care diagnosis, could contribute to racial and ethnic disparities.^16^ These factors are under more direct control of individual clinicians and health systems. Therefore, their contribution to health inequities could be addressed with short-term investment and attention.

In this study, we used electronic health record (EHR) data to quantify the relative contribution of clinical characteristics, public health determinants, treatment timing, and encounter-level care delivery factors to racial and ethnic disparities in COVID-19 treatment prescribing. Electronic health record data provide the opportunity to investigate the mechanisms of disparities at a more granular level of detail than research using other data sources, such as administrative claims data.^7,11,12^ We hypothesized that a significant portion of the racial and ethnic prescription gap may be associated with encounter-level factors. Results consistent with this hypothesis might support the investment of interventions and resources at the clinic level to address this disparity. Elucidating these mechanisms is a crucial first step toward guiding health system interventions to promote health equity in the midst of pandemic conditions.

## Methods

### Study Populations

This cross-sectional study used EHR data from a large academic health system in New England. The full cohort included any patient aged 18 years or older who received an order for a COVID-19 test in an outpatient clinic or emergency department between January 1, 2022, and January 31, 2024, up until their first positive test result (eMethods 1 in Supplement 1). The Mass General Brigham Institutional Review Board approved the study. Informed consent was not required because the research analyzed secondary data. The study followed the Strengthening the Reporting of Observational Studies in Epidemiology (STROBE) reporting guideline.

For the main analysis, we focused on a cohort of patients with positive COVID-19 test results, retaining only the first positive test result for patients with multiple positive results. We focused on patients with a positive test result to ensure that our sample population comprised individuals who should have reasonably been expected to be evaluated for outpatient treatment. We included descriptive statistics for individuals with freestanding prescriptions (ie, those prescribed without any documented test order or result) as presumed to have COVID-19 but excluded them from our main analyses given the limited information known about their care and treatment eligibility prior to prescription. We also limited the sample to outpatient practices with at least 25 documented COVID-19 test orders in the EHR during the study period to compare sites that were delivering some degree of consistent COVID-19 care over the 2-year period. The final sample included 530 clinical sites, the majority of which were internal medicine or family medicine primary practices or urgent care clinics.

### Study Outcomes

The primary outcome was receipt of an order of molnupiravir or nirmatrelvir within 7 days of positive COVID-19 test results (eMethods 2 in Supplement 1). These drugs comprised more than 95% of outpatient COVID-19 treatment received by the end of 2022.^11^ The data did not include information on prescription fills; thus, our analysis focused primarily on clinician intention to treat as demonstrated by an ordered prescription.

### Capturing Patient- and Clinic-Level Race and Ethnicity

This study used race and ethnicity data as listed in the EHR (self-reported or administrator observed and documented). In 2022, the health care system implemented a system-wide initiative to improve the accuracy and completeness of the race, ethnicity, and language data in the EHR. The program aimed to increase self-reporting of data through an outreach campaign to approximately 1 million adult primary care patients by both mail and patient portal messaging.^17,18^ The program ran as a pilot in 2021, was expanded in 2022, then ran for 2 more years before the data were accessed (2024). Though the program was presumed to increase the quality of available data, the race and ethnicity information in this study represent an unknown combination of self-reporting and administrator observation.

In our main results, we focused on presenting findings for Black and Latino patients, as they represent the largest underserved groups in the Boston, Massachusetts, area for whom we had sufficient data quality, granularity, and sample size to identify and conduct statistical inference analyses. All patients identified as Hispanic or Latino were categorized as such. Non-Hispanic Black patients were grouped as Black, and non-Hispanic White patients were grouped as White. Unadjusted results for patients identified as American Indian or Alaska Native, Asian or Pacific Islander, or multiracial were reported individually (eTable 1 in Supplement 1). These patients were grouped together into a non-Hispanic other category for regression analyses due to small subgroup sample sizes limiting careful statistical inference. Individuals who declined to give race or ethnicity or with missing information were included in analyses and categorized into an unknown category.

We also sorted primary and urgent care practices in our sample into quartiles based on the percentage of their total patient population (unique patients seen at a given practice) who were identified as Black or Latino. The highest quartile represented clinics that saw the highest percentage of underserved minority patients and inversely, for clinics in the lowest quartile. Results for unadjusted practice-level analyses present the mean rate observed across clinics in their respective quartiles.

### Other Patient Variables

Sex was categorized as reported in the EHR as male or female (individuals identifying as other than male or female were not included in the data). Insurance was categorized as commercial, Medicaid, Medicare, Medicare through dual eligibility or disability, none identified, or other (including government-provided, international, or worker’s compensation) (eMethods 3 in Supplement 1). Comorbidity status was determined using the Elixhauser Comorbidity Index, which was calculated from *International Statistical Classification of Diseases, Tenth Revision* codes associated with active conditions documented in the problem list.^19^ Age was recoded from a continuous variable into a categorical variable with 10-year age groups to allow for modeling of nonlinear trends. Patients with race-neutral estimated glomerular filtration rate^20,21^ less than 30 mL/min/1.73 m^2^, moderate to severe liver disease, or an active order for contraindicated medications were categorized as having a potential contraindication to treatment with nirmatrelvir (eMethods 4 in Supplement 1).^22,23^ Vaccination status was a binary indicator representing whether patients ever received any COVID-19 vaccine. Patients’ primary language documented in the EHR was recategorized as a binary variable for speaking English vs a different language.

### Clinical Encounter–Level Variables

We selected encounter-level care delivery characteristics that were under the direct influence of clinicians and/or the health care system at large (ie, that could be plausibly intervened upon). These characteristics included the type of test ordered or accepted by the clinician for COVID-19 diagnosis, virtual care use, and/or the clinic in which care was received. Virtual care usage was categorized as having a virtual visit by phone, video, or patient portal messaging. COVID-19 tests were characterized as in-clinic polymerase chain reaction, in-clinic rapid antigen, or patient-reported home antigen tests. Site of care was assigned as the clinic associated with the encounter in which a COVID-19 test order was placed or documented (eMethods 1 in Supplement 1).

### Statistical Analysis

Descriptive analyses were used to examine unadjusted differences in prescription order rates, clinical risk factors, and care characteristics, with bivariate statistical tests as appropriate. We then used nested linear probability models to examine differences in prescription likelihood, sequentially holding fixed clinical characteristics, public health, treatment timing, and encounter-level factors that we hypothesized had a meaningful association with the outcome (eMethods 5 in Supplement 1). Models estimated mean prescription likelihood across racial and ethnic groups, sequentially controlling for the aforementioned covariates. We created 6 nested models for preliminary examination of the influence of each of these covariate groups (eMethods 5 in Supplement 1).

We then applied the Gelbach decomposition method^24^ to quantify how much of the prescription gap across minoritized racial and ethnic groups was accounted for by inclusion of upstream (ie, clinical, public health determinant, and treatment timing) factors vs encounter-level care delivery factors. The Gelbach method uses the omitted variable bias equation^25^ to robustly partition the change in the coefficient of a variable of interest from the base model to the full model into unbiased estimates of how much of the observed gap is attributable to specific variables or groups of variables.^24,26^ We aggregated covariates into 6 categories, including clinical characteristics, public health determinants, epidemiologic timing, test type, visit type, and site of care, to understand which variables were the most important for influencing the racial and ethnic prescription gap (eMethods 5 in Supplement 1).

All models included robust variance estimators^27^ specifying departments as clusters. Two-sided statistical significance was assessed at *P* < .05. R, version 4.0 (R Foundation for Statistical Computing) and Stata, version 18 (StataCorp LLC) were used for the statistical analyses.

## Results

### Study Population

A total of 714 060 patients with EHR-documented COVID-19 test orders or freestanding medication orders were identified between January 2022 and January 2024, of whom 201 964 (28.3%; mean [SD] age, 54.0 [18.2] years; 64.6% female and 35.4% male; 0.1% identified as American Indian or Alaska Native, 3.7% as Black, 6.5% as Hispanic or Latino, 79.0% as White, 1.1% as other, and 4.7% as unknown race and ethnicity) had a first positive test result during the study period (Table 1; eTable 1 in Supplement 1). Among this cohort, 66.4% were commercially insured, 94.3% had English listed as their primary language, and 91.5% were vaccinated. The mean (SD) Elixhauser Comorbidity Index for patients with COVID-19 was 1.9 (2.0), and 16.7% had a potential contraindication to receiving nirmatrelvir. Descriptive statistics on rates of kidney disease, liver disease, and medication-based contraindications by race and ethnicity are reported in eTable 2 in Supplement 1.

**Table 1.** Cohort Clinical and Demographic Characteristics by Test Results and Prescription Outcomes^a^

Characteristic	Patients, No. (%)			
	Received COVID-19 test			Prescription without COVID-19 test results
	Patients with positive test results	No prescription after positive test results	Prescription after positive test results	
Patients, No. (row %)	201 964 (100)	141 971 (70.3)	59 993 (29.7)	39 359 (100)
Age, mean (SD), y	54.0 (18.2)	50.6 (18.3)	62.0 (15.5)	59.9 (15.5)
Sex				
Female	130 454 (64.6)	93 621 (65.9)	36 833 (61.4)	26 488 (67.3)
Male	71 510 (35.4)	48 350 (34.1)	23 160 (38.6)	12 871 (32.7)
Race and ethnicity				
American Indian or Alaska Native	275 (0.1)	211 (0.1)	64 (0.1)	92 (0.2)
Asian or Pacific Islander	9857 (4.9)	7309 (5.1)	2548 (4.2)	1766 (4.5)
Black	7508 (3.7)	5934 (4.2)	1574 (2.6)	1194 (3.0)
Hispanic or Latino	13 064 (6.5)	10 197 (7.2)	2867 (4.8)	2408 (6.1)
White	159 576 (79.0)	108 935 (76.7)	50 641 (84.4)	32 625 (82.9)
Other^b^	2244 (1.1)	1718 (1.2)	526 (0.9)	374 (1.0)
Unknown^c^	9440 (4.7)	7667 (5.4)	1773 (3.0)	900 (2.3)
Insurance coverage				
Commercial	134 075 (66.4)	99 207 (69.9)	34 868 (58.1)	23 879 (60.7)
Medicaid or Health Safety Net	13 932 (6.9)	10 930 (7.7)	3002 (5.0)	2464 (6.3)
Medicare	42 533 (21.1)	23 351 (16.4)	19 182 (32.0)	10 750 (27.3)
Medicare through dual eligibility or disability	7468 (3.7)	4977 (3.5)	2491 (4.2)	1959 (5.0)
None identified	3034 (1.5)	2734 (1.9)	300 (0.5)	201 (0.5)
Other^d^	922 (0.5)	772 (0.5)	150 (0.3)	106 (0.3)
Primary language				
English	190 525 (94.3)	132 818 (93.6)	57 707 (96.2)	38 039 (96.6)
Not English	11 439 (5.7)	9153 (6.4)	2286 (3.8)	1320 (3.4)
Elixhauser Comorbidity Index (calculated from the *ICD-10*), mean (SD)	1.9 (2.0)	1.7 (2.0)	2.5 (2.0)	2.9 (2.2)
Vaccination status				
Unvaccinated	17 182 (8.5)	14 405 (10.1)	2777 (4.6)	1193 (3.0)
Vaccinated	184 782 (91.5)	127 566 (89.9)	57 216 (95.4)	38 166 (97.0)
Contraindications for nirmatrelvir/ritonavir				
0	168 161 (83.3)	121 242 (85.4)	46 919 (78.2)	26 483 (67.3)
≥1^e^	33 803 (16.7)	20 729 (14.6)	13 074 (21.8)	12 876 (32.7)

Abbreviation: *ICD-10*, *International Statistical Classification of Diseases, Tenth Revision*.

^a^ Bivariate statistical tests for differences in prescription rates between racial and ethnic minority patients and White patients with positive COVID-19 test results were all statistically significant (full results of statistical tests for differences are reported in eTable 2 in Supplement 1).

^b^ Race or ethnicity was documented as other in the electronic health record system.

^c^ Included patients who declined to answer or for whom the field in the electronic health record was blank.

^d^ Other insurance coverage included government-provided insurance, international coverage, worker’s compensation, or motor vehicle accident coverage.

^e^ Contraindicated medication or comorbidity.

### Unadjusted Racial and Ethnic Disparities in COVID-19 Diagnosis Patterns and Treatment Rates

**Figure 1.**
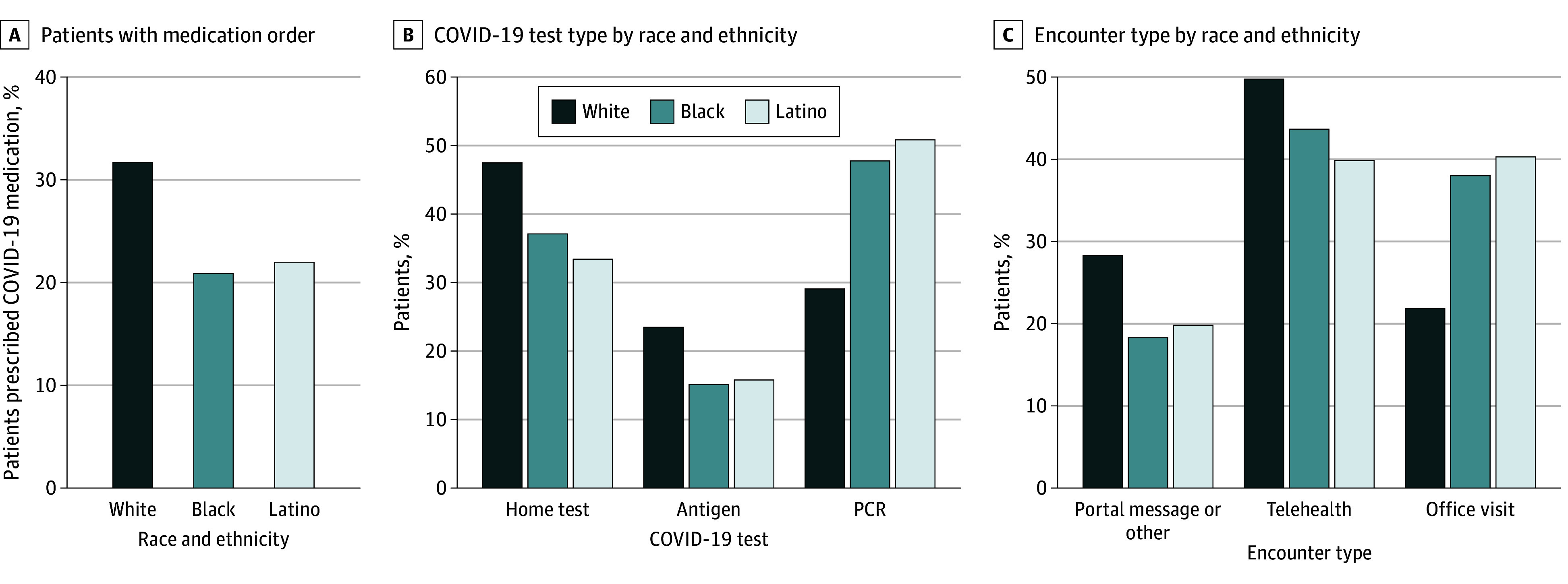
Medication Prescription Orders, Test Types, and Treatment Encounter Types Among Patients With Positive COVID-19 Test Results by Race and Ethnicity PCR indicates polymerase chain reaction.

Among patients with positive test results, 29.7% had a medication order placed. There were racial and ethnic disparities in unadjusted COVID-19 treatment rates between White patients (31.7% prescription rate) and those of all other racial and ethnic backgrounds, most notably Black (21.0% prescription rate) and Latino (21.9% prescription rate) (Figure 1). The largest differences and lowest prescription order rates were consistently observed among Black and Latino patients, with Black patients being 10.8 percentage points (95% CI, 9.7-11.8 percentage points; *P* < .001) less likely and Latino patients and 9.8 percentage points (95% CI, 9.0-10.6 percentage points; *P* < .001) less likely to receive prescription orders than White patients (eTables 1 and 3 in Supplement 1).

Overall, medication prescription rates increased from 24.9% of patients with positive tests in 2022 to 41.6% in 2023. Disparities in unadjusted medication prescription rates between Black patients and White patients decreased from −10.6 percentage points (95% CI, −11.8 to −9.5 percentage points) in 2022 to −8.4 percentage points (95% CI, −10.9 to −6.0 percentage points) in 2023 and for Latino patients, from −9.1 percentage points (95% CI, −9.9 to −8.2 percentage points) to −7.9 percentage points (95% CI, −9.8 to −6.0 percentage points). This trend was attributable to proportionally larger increases in prescription rates for Black and Latino patients than White patients over the 2-year time frame (eTable 4 in Supplement 1).

### Unadjusted Rates of Virtual Care Use and COVID-19 Test Type

Among racial and ethnic groups, White patients were most often treated through telehealth visits (49.8%) or patient portal messages (28.3%) and were diagnosed most often via home antigen tests (47.4%) (eTable 5 in Supplement 1). Black and Latino patients were less likely than White patients to have received virtual care (−16.2 percentage points [95% CI, −17.3 to −15.0 percentage points] and −18.4 percentage points [95% CI, −19.3 to −17.5 percentage points]), respectively) (both *P* < .001). Black and Latino patients were also less likely to have been diagnosed through clinician-ordered antigen tests (−8.4 percentage points [95% CI, −9.2 to −7.5 percentage points] and −7.7 percentage points [95% CI, −8.3 to −7.0 percentage points], respectively) tests or by home antigen tests (−10.3 percentage points [95% CI, −11.5 to −9.2 percentage points] and −14.1 percentage points [95% CI, −14.9 to −13.2 percentage points], respectively) than White patients (all *P* < .001) (Figure 1; eTables 3 and 5 in Supplement 1).

### Unadjusted Rates of COVID-19 Prescription Practices Across Clinics

**Figure 2.**
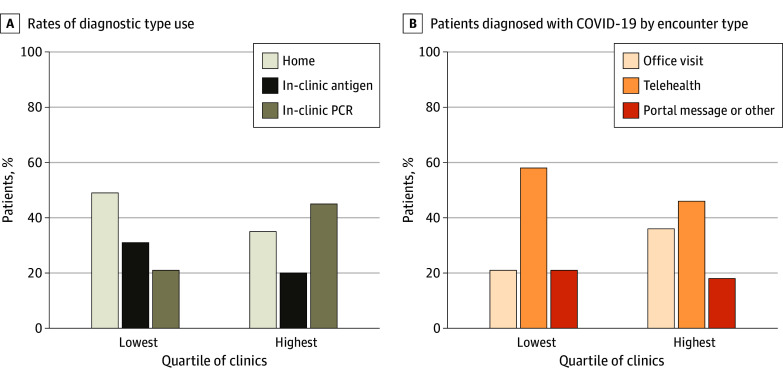
Test and Encounter Types Among Patients With Positive COVID-19 Test Results in the Highest and Lowest Quartiles of Clinics Serving Black and Latino Individuals The lowest quartile of clinics served 3% Black and/or Latino patients on average, and the highest quartile served 27% Black and/or Latino patients. PCR indicates polymerase chain reaction on average.

Clinics varied in their telehealth usage (mean [SD], 47% [32%]), home test use (mean [SD], 43% [29%]), and prescription order rates (mean [SD], 33% [17%]). The highest quartile of clinics serving the largest percentage of Black and Latino patients were 15.2 percentage points (95% CI, 14.6-15.8 percentage points; *P* < .001) more likely to see patients through an in-person encounter compared with clinics in the lowest quartile. Clinics in the highest quartile were also 24.8 percentage points (95% CI, 24.3-25.3 percentage points; *P* < .001) less likely to use home or clinic-based antigen tests for COVID-19 diagnosis and 8.2 percentage points (95% CI, 7.90-8.43 percentage points; *P* < .001) less likely to prescribe medication compared with clinics in the lowest quartile that served the fewest Black and Latino patients (eTable 6 in Supplement 1; Figure 2).

### Adjusted Models

Adjustment for clinical, public health, and treatment timing covariate groups (age, sex, comorbidities, contraindications, vaccination status, insurance, language, day of week, month, year) was associated with a decrease in prescription likelihood from −10.8 percentage points (95% CI, −11.8 to −9.7 percentage points; *P* < .001) to −3.3 percentage points (95% CI, −4.2 to −2.4 percentage points; *P* < .001) for Black patients and from −9.8 percentage points (95% CI, −10.6 to −9.0 percentage points; *P* < .001) to a nonsignificant finding of 0.7 percentage points (95% CI, 0.4-1.4 percentage points; *P* = .07) for Latino patients compared with White patients. After controlling for encounter-level characteristics (test type, visit type, and site of care), there was no significant disparity for Black patients (−0.6 percentage points [95% CI, −1.4 to 0.2 percentage points]; *P* = .14) and a small increase in prescription likelihood for Latino patients (0.8 percentage points [95% CI, 0.1-1.5 percentage points]; *P* = .01) (Table 2; eTable 7 in Supplement 1).

**Table 2.** Nested Modeling of Association Between Prescription Likelihood Across Racial and Ethnic Subgroups

Race and ethnicity	Estimated coefficient (95% CI)^a^						
	Unadjusted model	Clinical characteristics^b^	Public health determinants^c^	Time fixed effects^d^	Virtual care or visit type^e^	Test type^f^	Clinic or site of care^g^
Black	−0.108 (−0.118 to −0.097)^h^	−0.055 (−0.064 to −0.046)^h^	−0.053 (−0.062 to −0.044)^h^	−0.033 (−0.042 to −0.024)^h^	−0.030 (−0.039 to −0.021)^h^	−0.026 (−0.035 to −0.018)^h^	−0.006 (−0.014 to 0.002)
Hispanic or Latino	−0.098 (−0.106 to −0.090)^h^	−0.021 (−0.029 to −0.014)^h^	−0.011 (−0.019 to −0.004)^i^	0.007 (−0.000 to 0.014)	0.011 (0.004 to 0.019)^i^	0.015 (0.008 to 0.022)^h^	0.008 (0.001 to 0.015)^j^

^a^ Medications included nirmatrelvir/ritonavir and molnupiravir. Site of care refers to the clinic or practice in which the test order encounter or other previous encounter in the week before took place. The covariate coefficients in models represent the absolute percentage-point increase in prescription order likelihood compared with the specified reference group. White patients were the reference group for the estimates reported. The estimated coefficients for all covariates across models are reported in eTable 6 in Supplement 1. The sample size for all regression models was 201 964 patients.

^b^ Equivalent to the unadjusted model (ie, race and ethnicity) with the addition of age, sex, Elixhauser Comorbidity Index, and potential contraindications to treatment with nirmatrelvir/ritonavir covariates.

^c^ All covariates in the clinical characteristics model, as well as insurance, vaccination status, and an indicator for non-English primary language.

^d^ All covariates in the clinical characteristics and public health determinants models, as well as indicator variables for the visit year, month, and day of week.

^e^ All covariates in the clinical characteristics, public health determinants, and time fixed-effects models, as well as the visit type (office, telehealth, patient portal messaging, other).

^f^ All covariates in the clinical characteristics, public health determinants, time fixed-effects, and virtual care models, as well as diagnostic test type.

^g^ All covariates in the clinical characteristics, public health determinants, time fixed-effects, virtual care, and test type models, as well as indicator variables for the clinic or site of care.

^h^ *P* < .001.

^i^ *P* < .01.

^j^ *P* < .05.

### Quantifying Relative Factor Contributions to the Prescription Gap

**Figure 3.**
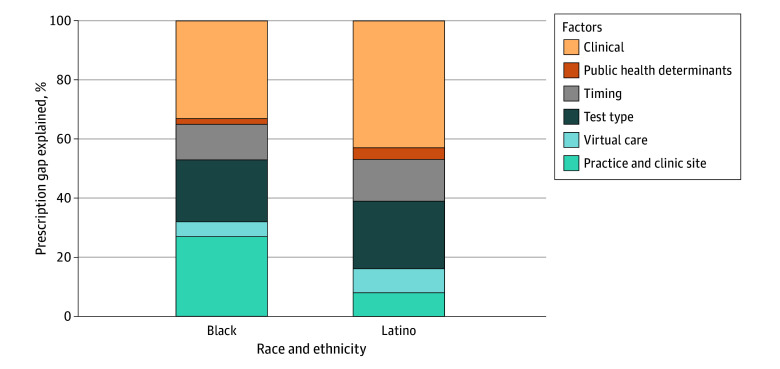
Gelbach Decomposition of the Relative Contribution of Factors Associated With the Racial and Ethnic Prescription Gap Among Patients With Positive COVID-19 Test Results Clinical factors included age, sex, comorbidities, and contraindications. Public health determinants included insurance, vaccination status, and primary language. Timing factors included day of the week, month, and year as fixed effects. Test types included in-clinic polymerase chain reaction, in-clinic antigen, and home tests. Virtual care factors included office visits, telehealth, and patient portal messaging.

For Black patients, 53% of the variation in prescription rates was explained by encounter-level characteristics, with 5% explained by differences in virtual care use, 21% by COVID-19 test type used, and 27% by the site of care. For Latino patients, 39% of variation in prescription rates was explained by encounter-level characteristics, with 8% explained by virtual care use, 23% by test type, and 8% by site of care (Figure 3; eTable 8 in Supplement 1).

## Discussion

In this cross-sectional study, we found a substantial COVID-19 prescription gap by race and ethnicity in a large academic health care system. Encounter-level characteristics explained approximately half of the disparity between Black and Latino patients and White patients, while clinical risk factors, public health determinants, and epidemiologic timing factors explained the other half. Wide variation across clinics in their use of home and clinician-ordered rapid antigen tests suggested that differential practice patterns and/or resources at various clinics influenced this disparity, providing actionable information to guide interventions by clinicians and practices to address this inequity. This study also offers a general approach for health care systems to better understand the factors underlying their local health disparities and create more targeted, community-specific interventions to address them in outpatient COVID-19 treatment or other areas of care.

The association between use of antigen tests and virtual care with disparities in prescribing may be related to their potential to decrease the time from symptom onset to diagnosis and treatment initiation. Home and rapid antigen tests contribute to quicker diagnoses, thereby increasing the potential benefit of oral treatment and the likelihood that a clinician places a prescription order. Rapid tests may also help avoid potential disruptions, such as having to make subsequent visits to laboratory sites for polymerase chain reaction testing or waiting 1 or more days for a test result and clinician review before medication prescription. Virtual care options may also play a substantial role for patients who experience delays in seeking care due to socioeconomic constraints, such as limited access to transportation or inflexible work schedules. Inequitable access to the technology needed for virtual visits may also create further disparities in prescribing rates. Virtual encounters may additionally influence prescribing behaviors, whether through increased clinician willingness to prescribe, differential acceptance of home test results, or changes in clinician-patient dynamics that may reduce the impact of visual implicit biases.

Structural racism may contribute to both health care access and delivery by influencing the resources available at different clinics and the ability of patients to navigate care. Clinics serving more historically marginalized populations may have fewer resources for rapid testing or virtual care, exacerbating racial inequities in timely treatment access. Differential access to these resources, whether it be cost, distribution, clinic-level infrastructure, or clinician behavior, could disproportionately put Black and Latino patients, who already face many other structural barriers in health care, at a disadvantage. Our findings also highlight that while a considerable portion of this disparity could be addressed by the health care system, full elimination of the disparity would probably require many large-scale interventions to minimize exposure to adverse social determinants of health and increase health justice at the population level.

Altogether, these findings have several implications for addressing this disparity in treatment access. First, clinic and clinician-level variation were significantly associated with inequities in prescription rates, suggesting that interventions at this level (particularly if targeted to clinics serving a high percentage of Black and Latino patients) may improve inequity in COVID-19 treatment. Second, the findings underscore the need for equitable access to rapid tests through decreased costs and logistic barriers surrounding home tests, increased rapid antigen test availability across clinic sites, and mitigation of any potential clinician bias in home test use. Campaigns to promote virtual care access may be particularly important for patients with delayed presentation for treatment relative to symptom onset.

### Limitations

This study has several limitations. First, the study focused mainly on patients who received a conclusive positive test result for COVID-19 and, therefore, did not investigate dynamics for symptomatic patients who did not have access to health care or who were not tested for COVID-19. In addition, the documentation of a positive home test result in the EHR may have been more likely if a clinician decided to prescribe antiviral treatment, which could introduce bias. Second, the EHR data contained information on prescription orders placed but not prescription fills; therefore, any disparities associated with inequitable pharmacy access were not captured. Third, we were unable to investigate patient and clinician knowledge, attitudes, and biases that might have affected prescription orders, as well as encounters in which a prescription offer was declined. Fourth, given the highly local nature of this disparity, the mechanisms presented in this study are not presumed to be generalizable; however, we aimed to provide an approach for other health systems interested in investigating influential care pathways contributing to disparities in their local environments. Finally, EHR data have limitations in terms of the quality and completeness of some demographic variables (importantly, race and ethnicity), comorbidities, and baseline health status information. While the data were relatively complete (5.1% missing), we were restricted in our analyses by limited sample sizes for several subgroups for whom racial and ethnic disparities in treatment exist. Though we were unable to conduct inference analyses for these groups, we reported unadjusted results for disaggregated groups in eTables 1 to 5 in Supplement 1.

## Conclusions

In this cross-sectional study, we observed a large racial and ethnic disparity in outpatient COVID-19 treatment in a single health care system and found that approximately half of the disparity was explained by encounter-level care delivery factors, such as access to rapid antigen tests and virtual care. The approach taken in this study may help inform targeted, local interventions to decrease disparities in the outpatient treatment of COVID-19 in individual health care systems. For example, intervention at the practice level, such as policies to increase accessibility of home tests, virtual visits, and patient portal messaging, may improve these disparities, particularly for individuals seen at clinics serving a high percentage of Black and Latino patients. This work also highlights the multifaceted upstream nature of this inequity. Half of the racial and ethnic prescription gap was explained by clinical, epidemiologic, and public health determinant factors, all of which are influenced by larger social and structural determinants of health and would require larger, longer-term intervention to decrease this disparity.

## Notes

Supplement 1. **eMethods 1.** Identifying the COVID-19–Positive Cohort, Test Orders, Test Results, and Visit Encounter Type **eMethods 2.** Identifying Medication Prescription Orders **eMethods 3.** Classifying Insurance Coverage **eMethods 4.** Identifying Comorbidities and Contraindications to COVID-19 Treatment **eMethods 5.** Estimator Variable Categories for Nested Models and the Gelbach Decomposition **eTable 1.** Full Cohort by Test Result and Prescription Outcome **eTable 2.** Kidney Disease, Liver Disease, and Medication Contraindication Cohorts **eTable 3.** Unadjusted Differences in Prescription Rates, Test Type Usage, and Virtual Care Use by Race and Ethnicity and Insurance Status **eTable 4.** Unadjusted Differences in Prescription Rates Over Time by Race and Ethnicity Subgroups **eTable 5.** Care Delivery Differences by Patients’ Race and Ethnicity and Insurance Coverage **eTable 6.** Department Variation in COVID-19 Test Usage, Virtual Care, and Prescription Rates **eTable 7.** Full Nested Linear Probability Models for Medication Prescription Order Receipt **eTable 8.** Gelbach Decomposition of Relative Factor Contribution to the Racial and Ethnic Prescription Order Gap

Supplement 2.
Data Sharing Statement
