# Supplementary material for: Racial Inequities and Access to COVID-19 Treatment
Source: JAMA Netw Open. 2025 Jul 1;8(7):e2518459. doi: 10.1001/jamanetworkopen.2025.18459 (PMC12215569; doi:10.1001/jamanetworkopen.2025.18459)
Supplement: Supplement 1. — eMethods 1. Identifying the COVID-19–Positive Cohort, Test Orders, Test Results, and Visit Encounter Type eMethods 2. Identifying Medication Prescription Orders eMethods 3. Classifying Insurance Coverage eMethods 4. Identifying Comorbidities and Contraindications to COVID-19 Treatment eMethods 5. Estimator Variable Categories for Nested Models and the Gelbach Decomposition eTable 1. Full Cohort by Test Result and Prescription Outcome eTable 2. Kidney Disease, Liver Disease, and Medication Contraindication Cohorts eTable 3. Unadjusted Differences in Prescription Rates, Test Type Usage, and Virtual Care Use by Race and Ethnicity and Insurance Status eTable 4. Unadjusted Differences in Prescription Rates Over Time by Race and Ethnicity Subgroups eTable 5. Care Delivery Differences by Patients’ Race and Ethnicity and Insurance Coverage eTable 6. Department Variation in COVID-19 Test Usage, Virtual Care, and Prescription Rates eTable 7. Full Nested Linear Probability Models for Medication Prescription Order Receipt eTable 8. Gelbach Decomposition of Relative Factor Contribution to the Racial and Ethnic Prescription Order Gap [file jamanetwopen-e2518459-s001.pdf]

Supplemental Online Content

Bromley-Dulfano R, Barnett ML. Racial inequities and access to COVID-19 treatment. *JAMA Netw Open*. 2025;8(6):e2517937. doi:10.1001/jamanetworkopen.2025.17937

eMethods 1. Identifying the COVID-19–Positive Cohort, Test Orders, Test Results, and Visit Encounter Type

eMethods 2. Identifying Medication Prescription Orders

eMethods 3. Classifying Insurance Coverage

eMethods 4. Identifying Comorbidities and Contraindications to COVID-19 Treatment

eMethods 5. Estimator Variable Categories for Nested Models and the Gelbach Decomposition

eTable 1. Full Cohort by Test Result and Prescription Outcome

eTable 2. Kidney Disease, Liver Disease, and Medication Contraindication Cohorts

eTable 3. Unadjusted Differences in Prescription Rates, Test Type Usage, and Virtual Care Use by Race and Ethnicity and Insurance Status

eTable 4. Unadjusted Differences in Prescription Rates Over Time by Race and Ethnicity Subgroups

eTable 5. Care Delivery Differences by Patients' Race and Ethnicity and Insurance Coverage

eTable 6. Department Variation in COVID-19 Test Usage, Virtual Care, and Prescription Rates

eTable 7. Full Nested Linear Probability Models for Medication Prescription Order Receipt

eTable 8. Gelbach Decomposition of Relative Factor Contribution to the Racial and Ethnic Prescription Order Gap

This supplemental material has been provided by the authors to give readers additional information about their work.

© 2025 Bromley-Dulfano R et al. *JAMA Network Open*.

eMethods 1. Identifying the COVID-19–Positive Cohort, Test Orders, Test Results, and Visit Encounter Type

Covid test order entries were identified through a general query search for relevant orders across the EHR database. We identified 47 test order types and manually classified order entries as either home antigen tests, clinic-based antigen tests, or clinic-based PCR tests. Test result information and encounter information were linked through an order ID and encounter ID number, respectively. Home antigen tests were identified through a standardized entry derived from providers manually entering patient-reported home test usage and the test result into the EHR. Data did not include the mechanism by which patients acquired a home test (e.g., self-bought, insurance-covered, COVIDTests.gov kits, test-to-treat sites).

Free-form text data from the raw test results were manually cleaned and recoded/classified as positive, negative, or incomplete/inconclusive. Ambiguous result text entries most often appeared to be intermediate/pending result notifications and were excluded from analysis. The encounter type was determined during the visit in which a covid test order was entered and were manually characterized as office visits, telehealth visits (including phone calls or video visits), or portal messages and other visits (e.g. nurse calls and messages). Only test orders (and their associated encounter) until a patient's first positive test (inclusive of this test and visit) were included in the dataset.

eMethods 2. Identifying Medication Prescriptions Orders

Medication order entries were similarly identified through a general query search for relevant orders across the EHR database. We identified 36 order entries for brand name or generic orders for molnupiravir and nirmatrelvir/ritonavir. Remdesivir was not included given its relative inaccessibility in requiring multiple in-person visits to an infusion center. A medication order was considered to be associated with a relevant positive COVID test if it was placed within 7 days of a Covid test order which resulted as positive. Medication prescription fill information was not visible within the data.

eMethods 3. Classifying Insurance Coverage

Medicare fee-for-service and Medicare Advantage coverage types were aggregated into a single Medicare category and dual eligible Medicare beneficiaries were disaggregated from this group. The Medicaid and Health Safety Net category included patients with base Medicaid coverage, those with Medicaid administered through Health Plans, and patients categorized in the EHR as covered under "Free Care". The category Other coverage included government provided insurance, international coverage, worker's compensation or Motor Vehicle accident coverage. Insurance coverage status was determined by the coverage that patients had at the beginning of the study period, January 1, 2022.

© 2025 Bromley-Dulfano R et al. *JAMA Network Open*.

eMethods 4. Identifying Comorbidities and Contraindications to COVID-19 Treatment
Comorbidity information was determined as an Elixhauser Comorbidity Index (ECI) score calculated directly from ICD-9 and ICD-10 codes associated with items on a patient's problem list that were marked "active." The score was calculated automatically by the *comorbidity* package in R.

Patients were considered to have a potential contraindication to treatment with nirmatrelvir if they met any of the following conditions:

- Having any historical eGFR test which resulted with a value of less than 30. If a dose-reduced treatment course with nirmatrelvir/ritonavir was possible (e.g., for patients with eGFR <60) patients were not flagged for a potential contraindication.
- Having severe hepatic impairment as determined under the same criteria (e.g. ICD codes) which identified patients with moderate to severe liver disease in the Elixhauser Comorbidity Index score calculation
- Having an active prescription order for any of the contraindicated medications listed in the National Institute of Health guidelines: https://www.covid19treatmentguidelines.nih.gov/therapies/antivirals-including-antibody-products/ritonavir-boosted-nirmatrelvir--paxlovid-/paxlovid-drug-drug-interactions/

Patients with any contraindication for treatment with nirmatrelvir were categorized into a separate group. Molnupiravir (recommended in cases where nirmatrelvir/ritonavir cannot be used) does not have the same contraindications, however, the presence of contraindications was still included in models given the potential for this factor to have created a differential barrier to treatment for these patients. Both the ECI and the contraindications variable include patients with moderate to severe liver disease (as identified through ICD diagnoses on Patients' problem lists) and patients with kidney disease (as identified through ICD diagnoses for the ECI and eGFR lab results for the contraindication indicator).

© 2025 Bromley-Dulfano R et al. *JAMA Network Open*.

eMethods 5. Estimator Variable Categories for Nested Models and the Gelbach Decomposition

We aggregated predictors into six categories: clinical composition, public health determinants, epidemiologic timing, test type, visit type, and site of care to understand which variables were the most important for influencing the racial prescription gap. The diagnostic test-type, virtual care use, and clinical site covariate groups were considered to be encounter-level factors (i.e., factors that may be amenable to immediate/short-term healthcare system and provider intervention). Other covariate groups were considered to be more upstream determinants that would requiring larger, long-term intervention and/or factors that were beyond the control of the healthcare system at the immediate level of the clinical encounter.

	**Upstream Structural Determinants**			**Encounter-Level Care Delivery Factors**		
**Covariate Group/Category**	**Clinical Characteristics**	**Public Health Determinants**	**Treatment Timing**	**COVID-19 Diagnostic Test Type**	**Visit Type/Virtual Care Use**	**Practice/Site of Care**
Variables included:	• Age<br>• Sex<br>• Elixhauser Comorbidity Index (ECI)<br>• Has Contra-indications	• Insurance<br>• Vaccination<br>• Language Spoken	• Day of Week<br>• Month<br>• Year	• In-Clinic PCR<br>• In-Clinic Antigen<br>• Home Antigen Test	• Office Visit<br>• Telephone or Video Visit<br>• e-Message or Other	• Clinic fixed-effects

© 2025 Bromley-Dulfano R et al. *JAMA Network Open*.

Supplemental Tables

eTable 1. Full Cohort by Test Result and Prescription Outcome

	Total	Received Covid Test			
		**First Positive**	**Negative**	**Inconclusive/ Incomplete**	**Freestanding Prescription**
**N (%)**	714,065 (100.0%)	201,964 (28.3%)	470,030 (65.8%)	2,707 (0.4%)	39,359 (5.5%)
**Age**					
18-29	100,154 (14.0%)	22,535 (11.2%)	75,854 (16.1%)	398 (14.7%)	1,367 (3.5%)
30-39	112,156 (15.7%)	30,871 (15.3%)	77,033 (16.4%)	545 (20.1%)	3,707 (9.4%)
40-49	96,854 (13.6%)	28,029 (13.9%)	63,634 (13.5%)	461 (17.0%)	4,730 (12.0%)
50-59	113,897 (16.0%)	34,190 (16.9%)	71,971 (15.3%)	393 (14.5%)	7,343 (18.7%)
60-69	135,264 (18.9%)	40,306 (20.0%)	83,873 (17.8%)	502 (18.5%)	10,583 (26.9%)
70-79	103,270 (14.5%)	31,073 (15.4%)	63,712 (13.6%)	292 (10.8%)	8,193 (20.8%)
80-89	43,428 (6.1%)	12,484 (6.2%)	27,829 (5.9%)	105 (3.9%)	3,010 (7.6%)
90+	9,042 (1.3%)	2,481 (1.2%)	6,124 (1.3%)	11 (0.4%)	426 (1.1%)
**Sex**					
Female	462,579 (64.8%)	130,454 (64.6%)	303,786 (64.6%)	1,850 (68.3%)	26,488 (67.3%)
Male	251,486 (35.2%)	71,510 (35.4%)	166,244 (35.4%)	857 (31.7%)	12,871 (32.7%)
**Race and Ethnicity**					
White	547,157 (76.6%)	159,576 (79.0%)	353,151 (75.1%)	1,802 (66.6%)	32,625 (82.9%)
Black	30,704 (4.3%)	7,508 (3.7%)	21,768 (4.6%)	234 (8.6%)	1,194 (3.0%)
Hispanic	53,985 (7.6%)	13,064 (6.5%)	38,205 (8.1%)	307 (11.3%)	2,408 (6.1%)
Asian or Pacific Islander	35,479 (5.0%)	9,857 (4.9%)	23,702 (5.0%)	154 (5.7%)	1,766 (4.5%)
American Indian or Alaska Native	1,036 (0.1%)	275 (0.1%)	666 (0.1%)	3 (0.1%)	92 (0.2%)
Other race	8,676 (1.2%)	2,244 (1.1%)	6,019 (1.3%)	39 (1.4%)	374 (1.0%)
Declined or Unavailable	37,028 (5.2%)	9,440 (4.7%)	26,519 (5.6%)	168 (6.2%)	900 (2.3%)
**Insurance Coverage Type**					
Commercial	465,252 (65.2%)	134,075 (66.4%)	305,491 (65.0%)	1,805 (66.7%)	23,879 (60.7%)
Medicaid or Health Safety Net	58,949 (8.3%)	13,932 (6.9%)	42,242 (9.0%)	310 (11.5%)	2,464 (6.3%)
Medicare	140,301 (19.6%)	42,533 (21.1%)	86,630 (18.4%)	387 (14.3%)	10,750 (27.3%)
Medicare | Dual Eligible or Disabled	33,624 (4.7%)	7,468 (3.7%)	24,084 (5.1%)	112 (4.1%)	1,959 (5.0%)
None Identified	11,303 (1.6%)	3,034 (1.5%)	7,991 (1.7%)	77 (2.8%)	201 (0.5%)
Other	4,636 (0.6%)	922 (0.5%)	3,592 (0.8%)	16 (0.6%)	106 (0.3%)
**Primary Language as Documented in EHR**					
English	664,620 (93.1%)	190,525 (94.3%)	433,598 (92.2%)	2,454 (90.7%)	38,039 (96.6%)
Language Other Than English	49,445 (6.9%)	11,439 (5.7%)	36,432 (7.8%)	253 (9.3%)	1,320 (3.4%)
**Elixhauser Comorbidity Index**	1.97 (2.16)	1.94 (2.01)	1.90 (2.21)	1.64 (1.97)	2.88 (2.18)
**Vaccination**					
Unvaccinated	83,488 (11.7%)	17,182 (8.5%)	64,793 (13.8%)	316 (11.7%)	1,193 (3.0%)
Vaccinated	630,577 (88.3%)	184,782 (91.5%)	405,237 (86.2%)	2,391 (88.3%)	38,166 (97.0%)
**Tests/Medication Paths**					
No Prescription Order	614,713 (86.1%)	141,976 (70.3%)	470,030 (100.0%)	2,707 (100.0%)	0 (0.0%)
Positive test + Prescription	59,993 (8.4%)	59,993 (29.7%)	0 (0.0%)	0 (0.0%)	0 (0.0%)
Freestanding Prescription	39,359 (5.5%)	0 (0.0%)	0 (0.0%)	0 (0.0%)	39,359 (100.0%)
**Has Contraindication to Nirmatrelvir/ Ritonavir**					
No	574,201 (80.4%)	168,161 (83.3%)	377,306 (80.3%)	2,249 (83.1%)	26,483 (67.3%)

© 2025 Bromley-Dulfano R et al. *JAMA Network Open*.

Yes (Has medication or comorbidity contraindication)	139,864 (19.6%)	33,803 (16.7%)	92,724 (19.7%)	458 (16.9%)	12,876 (32.7%)

The first column describes patient level clinical and sociodemographic characteristics of all patients who tested positive for COVID-19 or who received a medication order in our cohort. The second through fourth columns report characteristics (among patients with an EHR documented test) by test result. The fifth column represents the cohort of patients who received a prescription for COVID-19 treatment without an associated COVID test documented in the EHR. The category “Other Race or Ethnicity” includes patients whose race was listed as “Other” in the EHR.

© 2025 Bromley-Dulfano R et al. *JAMA Network Open*.

eTable 2. Kidney Disease, Liver Disease, and Medication Contraindication Cohorts

	Asian/Pacific Islander	American Indian/ Alaska Native	Black	Latino	Other	White	Unavailable	Total	Test
**N**	9,857 (4.9%)	275 (0.1%)	7,508 (3.7%)	13,064 (6.5%)	2,244 (1.1%)	159,576 (79.0%)	9,440 (4.7%)	N=201,964	
**Cohort Flagged as Having Contraindication to Treatment with Nirmatrelvir/Ritonavir, By Contraindication**									
No	8,700 (88.3%)	231 (84.0%)	6,298 (83.9%)	11,202 (85.7%)	1,909 (85.1%)	131,032 (82.1%)	8,789 (93.1%)	168,161 (83.3%)	<0.001
Yes	1,157 (11.7%)	44 (16.0%)	1,210 (16.1%)	1,862 (14.3%)	335 (14.9%)	28,544 (17.9%)	651 (6.9%)	33,803 (16.7%)	
**Takes Contraindicated Medication**									
No	8,714 (88.4%)	231 (84.0%)	6,319 (84.2%)	11,233 (86.0%)	1,918 (85.5%)	131,418 (82.4%)	8,793 (93.1%)	168,626 (83.5%)	<0.001
Yes	1,143 (11.6%)	44 (16.0%)	1,189 (15.8%)	1,831 (14.0%)	326 (14.5%)	28,158 (17.6%)	647 (6.9%)	33,338 (16.5%)	
**eGFR <30 mL/min/1.73 m2**									
No	9,836 (99.8%)	-	7,475 (99.6%)	13,029 (99.7%)	-	159,200 (99.8%)	-	201,489 (99.8%)	<0.001
Yes	21 (0.2%)	-	33 (0.4%)	35 (0.3%)	-	376 (0.2%)	-	475 (0.2%)	
**ICD-Based Moderate to Severe Liver Disease**									
No	-	-	7,496 (99.8%)	13,039 (99.8%)	-	159,260 (99.8%)	-	201,597 (99.8%)	<0.001
Yes	-	-	12 (0.2%)	25 (0.2%)	-	316 (0.2%)	-	367 (0.2%)	
**Patients Identified With Liver and Kidney Disease (Mild to Severe Disease)**									
**eGFR <60 mL/min/1.73 m2**									
No	9,772 (99.1%)	-	7,307 (97.3%)	12,895 (98.7%)	2,214 (98.7%)	156,079 (97.8%)	9,384 (99.4%)	197,922 (98.0%)	<0.001
Yes	85 (0.9%)	-	201 (2.7%)	169 (1.3%)	30 (1.3%)	3,497 (2.2%)	56 (0.6%)	4,042 (2.0%)	
**ICD-Based Kidney Disease**									
No	9,754 (99.0%)	-	7,348 (97.9%)	12,923 (98.9%)	2,216 (98.8%)	155,292 (97.3%)	9,336 (98.9%)	197,141 (97.6%)	<0.001
Yes	103 (1.0%)	-	160 (2.1%)	141 (1.1%)	28 (1.2%)	4,284 (2.7%)	104 (1.1%)	4,823 (2.4%)	
**ICD-Based Liver Disease**									
No	9,626 (97.7%)	-	7,399 (98.5%)	12,689 (97.1%)	2,198 (98.0%)	155,814 (97.6%)	9,347 (99.0%)	197,339 (97.7%)	<0.001
Yes	231 (2.3%)	-	109 (1.5%)	375 (2.9%)	46 (2.0%)	3,762 (2.4%)	93 (1.0%)	4,625 (2.3%)	

Patients were considered to have a contraindication to nirmatrelvir/ritonavir if switching to another medication for treatment (e.g. molnupiravir) was recommended. Groups were not necessarily mutually exclusive (e.g., a patient could have kidney, liver, and medicine contraindications). Patients with eGFR<30 were included as a subset of those with eGFR<60 (and likely also ICD-based kidney disease, if documented in the Problem List). ICD-based diagnoses were drawn from EHR Problem Lists and may represent underestimates of true prevalence of these conditions. More details on these criteria can be found in eMethods 4. The “Other race or ethnicity” category comprises patients whose race or ethnicity was documented as “Other” in the EHR system.

© 2025 Bromley-Dulfano R et al. *JAMA Network Open*.

eTable 3. Unadjusted Differences in Prescription Rates, Test Type Usage, and Virtual Care Use by Race and Ethnicity and Insurance Status

	Prescription Rates	In-clinic Rapid Ag Test Use	Home Test Use	Any Rapid Antigen Use (Home or In-clinic)	Virtual Care Use
**Race and Ethnicity**					
**Black**	-0.108***	-0.084***	-0.103***	-0.187***	-0.162***
	[-0.118,-0.097]	[-0.092,-0.075]	[-0.115,-0.092]	[-0.198,-0.175]	[-0.173,-0.150]
**Hispanic or Latino**	-0.098***	-0.077***	-0.141***	-0.217***	-0.184***
	[-0.106,-0.090]	[-0.083,-0.070]	[-0.149,-0.132]	[-0.226,-0.209]	[-0.193,-0.175]
**Other Race or Ethnicity**	-0.064***	-0.054***	-0.038***	-0.092***	-0.092***
	[-0.072,-0.056]	[-0.061,-0.047]	[-0.047,-0.029]	[-0.101,-0.083]	[-0.101,-0.084]
**Unknown**	-0.130***	-0.085***	-0.079***	-0.164***	-0.180***
	[-0.138,-0.121]	[-0.093,-0.078]	[-0.089,-0.069]	[-0.174,-0.154]	[-0.190,-0.170]
**Insurance**					
**Medicaid or Health Safety Net**	-0.045***	-0.043***	-0.128***	-0.170***	-0.131***
	[-0.052,-0.037]	[-0.049,-0.036]	[-0.136,-0.119]	[-0.179,-0.162]	[-0.139,-0.122]
**Medicare**	0.191***	0.057***	0.045***	0.102***	0.097***
	[0.186,0.196]	[0.052,0.062]	[0.040,0.051]	[0.098,0.107]	[0.093,0.101]
**Medicare - Dual Eligible or Disabled**	0.073***	0.022***	-0.053***	-0.031***	0.021***
	[0.063,0.084]	[0.012,0.031]	[-0.064,-0.041]	[-0.042,-0.020]	[0.011,0.030]
**None Identified**	-0.161***	-0.034***	-0.037***	-0.071***	-0.104***
	[-0.172,-0.150]	[-0.048,-0.021]	[-0.054,-0.019]	[-0.089,-0.053]	[-0.122,-0.087]
**Other**	-0.097***	-0.033*	-0.086***	-0.118***	-0.165***
	[-0.121,-0.073]	[-0.057,-0.008]	[-0.117,-0.054]	[-0.150,-0.086]	[-0.197,-0.133]

We report 95% confidence intervals in brackets; * p<0.05, ** p<0.01, *** p<0.001 for Wald tests for differences in samples (which given our sample size approximate t-tests). The covariate coefficients in models represent the absolute percentage point changes in prescription order, test order, or virtual care use likelihood relative to the specified reference group. White patients and commercially insured patients were the reference groups for their respective covariates. The category "Other Race or Ethnicity" includes American Indian or Alaskan Native, Asian/Pacific Islander, multi-racial and/or patients whose race was listed as "Other" in the EHR. Sample size for all regression models was N=201,964.

© 2025 Bromley-Dulfano R et al. *JAMA Network Open*.

eTable 4. Unadjusted Differences in Prescription Rates Over Time by Race and Ethnicity Subgroups

**Average Annual Prescription Rate**	**2022**	**2023**
All COVID-19 positive patients	24.9%	41.6%
**Average Unadjusted Difference in Prescription Rates Relative to White Patients**		
Black	-0.106***	-0.084***
	[-0.118,-0.095]	[-0.109,-0.060]
Latino	-0.091***	-0.079***
	[-0.099,-0.082]	[-0.098,-0.060]
Other Race or Ethnicity	-0.067***	-0.041***
	[-0.077,-0.058]	[-0.059,-0.023]
Unknown	-0.120***	-0.141***
	[-0.131,-0.109]	[-0.161,-0.122]

We report 95% confidence intervals in brackets; * p<0.05, ** p<0.01, *** p<0.001 for Wald tests for differences in samples (which given our sample size approximate t-tests). The covariate coefficients in models represent the average absolute percentage point change in prescription order likelihood across race and ethnicity subgroups relative to White patients. The category “Other Race or Ethnicity” includes American Indian or Alaskan Native, Asian/Pacific Islander, multi-racial and/or patients whose race was listed as “Other” in the EHR. The sample size was N=201,964.

© 2025 Bromley-Dulfano R et al. *JAMA Network Open*.

eTable 5. Care Delivery Differences By Patients' Race and Ethnicity and Insurance Coverage

Race and Ethnicity

	White	Black	Latino	API	AI/AN	Other	Missing
**N**	159,576 (79.0%)	7,508 (3.7%)	13,064 (6.5%)	9,857 (4.9%)	275 (0.1%)	2,244 (1.1%)	9,440 (4.7%)
**Test Encounter Type**							
Office visit	34,961 (21.9%)	2,858 (38.1%)	5,265 (40.3%)	2,986 (30.3%)	90 (32.7%)	778 (34.7%)	3,764 (39.9%)
Telehealth	79,437 (49.8%)	3,277 (43.6%)	5,210 (39.9%)	4,575 (46.4%)	122 (44.4%)	943 (42.0%)	4,225 (44.8%)
e-Message or Other	45,178 (28.3%)	1,373 (18.3%)	2,589 (19.8%)	2,296 (23.3%)	63 (22.9%)	523 (23.3%)	1,451 (15.4%)
**COVID Test Type**							
PCR	46,382 (29.1%)	3,586 (47.8%)	6,638 (50.8%)	3,639 (36.9%)	104 (37.8%)	995 (44.3%)	4,292 (45.5%)
Antigen	37,493 (23.5%)	1,137 (15.1%)	2,066 (15.8%)	1,803 (18.3%)	55 (20.0%)	380 (16.9%)	1,413 (15.0%)
Home Test	75,701 (47.4%)	2,785 (37.1%)	4,360 (33.4%)	4,415 (44.8%)	116 (42.2%)	869 (38.7%)	3,735 (39.6%)

Insurance Coverage

	Commercial	Medicaid or Safety Net	Medicare	Medicare Dual Eligible or Disabled	None Identified	Other
**N**	134,075 (66.4%)	13,932 (6.9%)	42,533 (21.1%)	7,468 (3.7%)	3,034 (1.5%)	922 (0.5%)
**Test Encounter Type**						
Office visit	34,975 (26.1%)	5,453 (39.1%)	6,978 (16.4%)	1,795 (24.0%)	1,108 (36.5%)	393 (42.6%)
Telehealth	64,008 (47.7%)	5,326 (38.2%)	23,089 (54.3%)	3,463 (46.4%)	1,541 (50.8%)	362 (39.3%)
e-Message or Other	35,092 (26.2%)	3,153 (22.6%)	12,466 (29.3%)	2,210 (29.6%)	385 (12.7%)	167 (18.1%)
**COVID Test Type**						
PCR	44,514 (33.2%)	7,000 (50.2%)	9,772 (23.0%)	2,712 (36.3%)	1,223 (40.3%)	415 (45.0%)
Antigen	28,210 (21.0%)	2,335 (16.8%)	11,372 (26.7%)	1,732 (23.2%)	534 (17.6%)	164 (17.8%)
Home Test	61,351 (45.8%)	4,597 (33.0%)	21,389 (50.3%)	3,024 (40.5%)	1,277 (42.1%)	343 (37.2%)

The table shows the average crude rates of diagnostic test type used and encounter visit type utilized for COVID-19 positive patients in our cohort. The “Other” category includes patients whose race or ethnicity was listed as “Other” in the EHR. The missing race category includes patients with no data listed in the EHR and patients who were listed as having declined to answer. All estimates are listed as n (%).

© 2025 Bromley-Dulfano R et al. *JAMA Network Open.*

eTable 6. Department Variation in COVID-19 Test Usage, Virtual Care, and Prescription Rates

Average Rates in Clinic Quartile	Quartile 1 (n= 28,104, 3% Black and Latino)	Quartile 2 (n= 33,430, 6% Black and Latino)	Quartile 3 (n= 46,007, 11% Black and Latino)	Quartile 4 (n= 37,667, 27% Black and Latino)
**Test Type Frequency**				
% Home Test Use Rate	0.486	0.391	0.499	0.349
95%CI	0.483, 0.49	0.388, 0.394	0.496, 0.502	0.346, 0.351
% Clinic-Based PCR Test	0.207	0.331	0.295	0.455
95%CI	0.204, 0.210	0.327, 0.335	0.292, 0.298	0.451, 0.459
% Clinic-Based Ag Test	0.307	0.278	0.206	0.196
95%CI	0.304, 0.309	0.276, 0.280	0.204, 0.207	0.194, 0.198
**Encounter Type Frequency**				
% Office Visits	0.209	0.308	0.271	0.361
95%CI	0.205, 0.213	0.303, 0.312	0.267, 0.275	0.357, 0.365
% Telehealth Visits	0.58	0.447	0.44	0.459
95%CI	0.577, 0.584	0.444, 0.451	0.437, 0.442	0.455, 0.463
% Portal Messaging or Other	0.211	0.245	0.289	0.18
95%CI	0.486,0.213	0.243, 0.247	0.287, 0.291	0.178, 0.182
**Prescription Rates**				
Positivity Rate (Among Clinic-Based Tests)	0.566	0.5	0.489	0.447
95%CI	0.563, 0.57	0.497, 0.503	0.486, 0.491	0.443, 0.45
Prescription Rate	0.379	0.305	0.351	0.297
95%CI	0.377, 0.381	0.303, 0.307	0.35, 0.352	0.296, 0.299

The table shows the average rate of various test types used, encounter visit types, test positivity rate, and prescription rates across clinic quartiles, divided by the % of Black and Latino patients served within practices. Only primary care and urgent care practices were included for this sub-analysis (outpatient specialty practices were excluded). For example, clinics in the fourth quartile, on average, served a 27% Black and Latino patient population, offered home tests roughly 35% of the time, saw patients through office visits for Covid approximately 36% of the time and prescribed treatment about 30% of the time.

© 2025 Bromley-Dulfano R et al. *JAMA Network Open*.

eTable 7. Full Nested Linear Probability Models for Medication Prescription Order Receipt

	Unadjusted Model	Clinical Characteristics	Public Health Determinants	Time Fixed Effects	Virtual Care/Visit Type	Test Type	Clinic/Site of Care
**Race and Ethnicity**							
Black	-0.108***	-0.055***	-0.053***	-0.033***	-0.030***	-0.026***	-0.006
	[-0.118,-0.097]	[-0.064,-0.046]	[-0.062,-0.044]	[-0.042,-0.024]	[-0.039,-0.021]	[-0.035,-0.018]	[-0.014,0.002]
Hispanic or Latino	-0.098***	-0.021***	-0.011**	0.007	0.011**	0.015***	0.008*
	[-0.106,-0.090]	[-0.029,-0.014]	[-0.019,-0.004]	[-0.000,0.014]	[0.004,0.019]	[0.008,0.022]	[0.001,0.015]
Other	-0.064***	0.009*	0.011**	0.003	0.007	0.008*	0.012***
	[-0.072,-0.055]	[0.002,0.017]	[0.003,0.019]	[-0.004,0.011]	[-0.000,0.014]	[0.001,0.016]	[0.005,0.018]
Unknown	-0.130***	-0.035***	0	-0.014**	-0.015**	-0.014**	0.007
	[-0.139,-0.120]	[-0.043,-0.027]	[-0.009,0.010]	[-0.024,-0.005]	[-0.024,-0.006]	[-0.023,-0.005]	[-0.002,0.015]
**Age**							
30-39		0.057***	0.052***	0.048***	0.040***	0.037***	0.029***
		[0.052,0.063]	[0.047,0.058]	[0.043,0.053]	[0.035,0.045]	[0.032,0.042]	[0.024,0.034]
40-49		0.127***	0.122***	0.115***	0.107***	0.104***	0.067***
		[0.121,0.133]	[0.115,0.128]	[0.109,0.121]	[0.101,0.113]	[0.098,0.110]	[0.061,0.072]
50-59		0.200***	0.194***	0.174***	0.165***	0.161***	0.108***
		[0.194,0.206]	[0.188,0.200]	[0.168,0.181]	[0.159,0.171]	[0.155,0.168]	[0.102,0.114]
60-69		0.285***	0.276***	0.244***	0.231***	0.227***	0.160***
		[0.278,0.291]	[0.269,0.283]	[0.237,0.250]	[0.225,0.238]	[0.220,0.234]	[0.154,0.167]
70-79		0.332***	0.316***	0.275***	0.260***	0.256***	0.184***
		[0.324,0.339]	[0.307,0.325]	[0.266,0.283]	[0.251,0.269]	[0.248,0.265]	[0.176,0.192]
80-89		0.325***	0.310***	0.269***	0.252***	0.251***	0.179***
		[0.315,0.336]	[0.298,0.322]	[0.257,0.280]	[0.241,0.264]	[0.240,0.263]	[0.168,0.190]
90+		0.271***	0.258***	0.228***	0.207***	0.209***	0.139***
		[0.250,0.291]	[0.237,0.280]	[0.208,0.249]	[0.187,0.228]	[0.189,0.230]	[0.120,0.159]
**Male Sex**		0.009***	0.011***	0.016***	0.021***	0.022***	0.005**
		[0.005,0.013]	[0.007,0.015]	[0.012,0.020]	[0.017,0.025]	[0.018,0.026]	[0.002,0.009]
**Elixhauser**		0.013***	0.013***	0.014***	0.014***	0.015***	0.010***
		[0.012,0.015]	[0.012,0.014]	[0.013,0.015]	[0.013,0.016]	[0.014,0.016]	[0.009,0.011]
**Has Contraindication**		0.028***	0.026***	0.004	0.006*	0.005	0.012***
		[0.022,0.034]	[0.020,0.032]	[-0.001,0.010]	[0.001,0.012]	[-0.001,0.010]	[0.007,0.017]
**Insurance Coverage**							
Medicaid or Health Care Safety Net			0.013***	0.032***	0.036***	0.039***	0.007
			[0.006,0.021]	[0.025,0.039]	[0.029,0.043]	[0.032,0.046]	[-0.000,0.013]
Medicare			0.014***	0.009*	0.009*	0.009*	0.006
			[0.006,0.021]	[0.001,0.016]	[0.002,0.017]	[0.002,0.016]	[-0.001,0.013]
Medicare – Dual Eligible or Disabled			-0.039***	-0.032***	-0.028***	-0.024***	-0.032***
			[-0.050,-0.027]	[-0.043,-0.020]	[-0.039,-0.017]	[-0.035,-0.013]	[-0.042,-0.021]
None Identified			-0.059***	-0.057***	-0.074***	-0.077***	0.010*
			[-0.070,-0.048]	[-0.068,-0.046]	[-0.085,-0.062]	[-0.088,-0.065]	[0.000,0.020]
Other			-0.092***	-0.081***	-0.076***	-0.077***	-0.026*
			[-0.115,-0.068]	[-0.105,-0.058]	[-0.099,-0.052]	[-0.100,-0.053]	[-0.047,-0.005]
**Primary Language Not English**			-0.040***	-0.030***	-0.025***	-0.022***	-0.006
			[-0.048,-0.031]	[-0.038,-0.021]	[-0.033,-0.016]	[-0.031,-0.014]	[-0.014,0.002]
**Vaccinated**			0.085***	0.072***	0.044***	0.042***	0.036***
			[0.079,0.091]	[0.066,0.078]	[0.038,0.050]	[0.036,0.047]	[0.030,0.042]

© 2025 Bromley-Dulfano R et al. *JAMA Network Open*.

**Month**							
February				0.086***	0.071***	0.062***	0.062***
				[0.077,0.095]	[0.062,0.080]	[0.053,0.071]	[0.054,0.071]
March				0.126***	0.110***	0.100***	0.092***
				[0.116,0.136]	[0.100,0.120]	[0.090,0.111]	[0.082,0.101]
April				0.204***	0.186***	0.174***	0.157***
				[0.196,0.212]	[0.178,0.194]	[0.166,0.182]	[0.149,0.165]
May				0.233***	0.209***	0.192***	0.170***
				[0.226,0.239]	[0.203,0.216]	[0.185,0.198]	[0.164,0.176]
June				0.227***	0.202***	0.185***	0.160***
				[0.219,0.235]	[0.194,0.210]	[0.177,0.193]	[0.153,0.168]
July				0.237***	0.214***	0.194***	0.165***
				[0.229,0.245]	[0.206,0.222]	[0.186,0.203]	[0.158,0.173]
August				0.231***	0.206***	0.188***	0.160***
				[0.223,0.239]	[0.198,0.214]	[0.180,0.196]	[0.152,0.167]
September				0.216***	0.192***	0.174***	0.144***
				[0.208,0.224]	[0.184,0.200]	[0.166,0.182]	[0.136,0.151]
October				0.194***	0.171***	0.154***	0.126***
				[0.185,0.202]	[0.163,0.179]	[0.146,0.162]	[0.119,0.134]
November				0.181***	0.163***	0.147***	0.122***
				[0.172,0.190]	[0.154,0.172]	[0.139,0.156]	[0.114,0.130]
December				0.214***	0.195***	0.179***	0.143***
				[0.207,0.221]	[0.188,0.202]	[0.172,0.185]	[0.136,0.149]
**Day of Week**							
Tuesday				-0.065***	-0.061***	-0.060***	-0.039***
				[-0.073,-0.056]	[-0.069,-0.053]	[-0.068,-0.052]	[-0.046,-0.031]
Wednesday				-0.057***	-0.053***	-0.053***	-0.031***
				[-0.066,-0.049]	[-0.062,-0.045]	[-0.061,-0.044]	[-0.038,-0.023]
Thursday				-0.048***	-0.047***	-0.045***	-0.020***
				[-0.056,-0.039]	[-0.055,-0.038]	[-0.053,-0.037]	[-0.027,-0.012]
Friday				-0.038***	-0.037***	-0.035***	-0.018***
				[-0.047,-0.029]	[-0.045,-0.028]	[-0.044,-0.027]	[-0.025,-0.010]
Saturday				-0.039***	-0.039***	-0.038***	-0.019***
				[-0.047,-0.030]	[-0.048,-0.030]	[-0.047,-0.030]	[-0.027,-0.011]
Sunday				0.011*	0.016**	0.018***	0.003
				[0.001,0.022]	[0.006,0.026]	[0.008,0.028]	[-0.006,0.012]
**Year**							
2023				0.149***	0.136***	0.127***	0.085***
				[0.144,0.154]	[0.131,0.140]	[0.122,0.132]	[0.081,0.090]
2024				0.249***	0.228***	0.210***	0.151***
				[0.239,0.260]	[0.218,0.239]	[0.199,0.221]	[0.141,0.161]
**Encounter Type**							
Telehealth					0.134***	0.060***	0.123***
					[0.129,0.138]	[0.054,0.066]	[0.112,0.135]
e-Message or Other					0	-0.058***	-0.003
					[-0.004,0.005]	[-0.064,-0.053]	[-0.015,0.008]
**Test Type**							
Antigen						0.076***	0.065***
						[0.070,0.083]	[0.058,0.072]
Home Test						0.103***	0.110***
						[0.097,0.108]	[0.103,0.116]
**Constant**	0.317***	0.076***	0.006	-0.130***	-0.144***	-0.138***	-0.107***
	[0.315,0.320]	[0.072,0.081]	[-0.001,0.012]	[-0.140,-0.120]	[-0.154,-0.135]	[-0.148,-0.129]	[-0.119,-0.095]

© 2025 Bromley-Dulfano R et al. *JAMA Network Open*.

We report 95% confidence intervals in brackets; * p<0.05, ** p<0.01, *** p<0.001. The covariate coefficients in models represent the absolute percentage point increase in prescription order likelihood relative to the specified reference group. The sample size was N=201,964 for all models.

© 2025 Bromley-Dulfano R et al. *JAMA Network Open*.

eTable 8. Gelbach Decomposition of Relative Factor Contribution to the Racial and Ethnic Prescription Order Gap

Black Patients	Estimate	95% Confidence Interval		% of Gap Explained
Clinical (Age, Comorbidities, Contraindications)	-0.034	-0.037	-0.032	33%
Social/Public Health (Insurance, Vaccination, Language)	-0.002	-0.004	0	2%
Encounter Type (Office, Telehealth, Portal/Other)	-0.005	-0.007	-0.003	5%
Test Type (Home, Clinic Ag, Clinic PCR)	-0.022	-0.023	-0.02	21%
Practice/Clinic Site	-0.028	-0.033	-0.024	27%
Treatment Timing (Day of week, Month, Year)	-0.012	-0.014	-0.011	12%
Total Amount of Prescription Variation Explained By Covariates Above	-0.103	-0.11	-0.099	-
**Latino Patients**	**Estimate**	**95% Confidence Interval**		**% of Gap Explained**
Clinical (Age, Comorbidities, Contraindications)	-0.045	-0.047	-0.042	43%
Social/Public Health (Insurance, Vaccination, Language)	-0.004	-0.007	-0.001	4%
Encounter Type (Office, Telehealth, Portal/Other)	-0.008	-0.01	-0.006	8%
Test Type (Home, Clinic Ag, Clinic PCR)	-0.024	-0.026	-0.023	23%
Practice/Clinic Site	-0.008	-0.013	-0.005	8%
Treatment Timing (Day of week, Month, Year)	-0.015	-0.016	-0.014	14%
Total Amount of Prescription Variation Explained By Covariates Above	-0.104	-0.109	-0.099	-

© 2025 Bromley-Dulfano R et al. *JAMA Network Open*.
