# Supplementary material for: Racial Inequities and Access to COVID-19 Treatment
Source: JAMA Netw Open. 2025 Jul 1;8(7):e2518459. doi: 10.1001/jamanetworkopen.2025.18459 (PMC12215569; doi:10.1001/jamanetworkopen.2025.18459)
Supplement: Supplement 2. — Data Sharing Statement [file jamanetwopen-e2518459-s002.pdf]

Data Sharing Statement

Bromley-Dulfano. Racial Inequities and Access to COVID-19 Treatment. *JAMA Netw Open*. Published July 01, 2025. doi:10.1001/jamanetworkopen.2025.18459

Data
Data available: No

Additional Information
Explanation for why data not available: The data are confidential electronic health record data and as such cannot be made available.
